# Spatial characterization of colonies of the flying fox bat, a carrier of Nipah Virus in Thailand

**DOI:** 10.1186/s12917-015-0390-0

**Published:** 2015-03-28

**Authors:** Weerapong Thanapongtharm, Catherine Linard, Witthawat Wiriyarat, Pornpiroon Chinsorn, Budsabong Kanchanasaka, Xiangming Xiao, Chandrashekhar Biradar, Robert G Wallace, Marius Gilbert

**Affiliations:** Department of Livestock Development (DLD), Bangkok, Thailand; Lutte biologique et Ecologie spatiale (LUBIES), Université Libre de Bruxelles, Brussels, Belgium; Fonds National de la Recherche Scientifique (FNRS), Brussels, Belgium; The Monitoring and Surveillance Center for Zoonotic Diseases in Wildlife and Exotic Animals (MOZWE), Mahidol University, Nakhonpatom, Thailand; Department of National Parks, Wildlife, and Plant Conservation, Bangkok, Thailand; Department of Microbiology and Plant Biology, Center for Spatial Analysis, University of Oklahoma, Norman, OK 73019 USA; Institute of Biodiversity Science, Fudan University, Shanghai, 200433 China; International Center for Agricultural Research in Dry Areas (ICARDA), Amman, Jordan; Institute for Global Studies, University of Minnesota, Minneapolis, USA

**Keywords:** Flying foxes, Nipah, Species distribution model, Ensemble modeling, Potential surface analysis

## Abstract

**Background:**

A major reservoir of Nipah virus is believed to be the flying fox genus *Pteropus*, a fruit bat distributed across many of the world’s tropical and sub-tropical areas. The emergence of the virus and its zoonotic transmission to livestock and humans have been linked to losses in the bat’s habitat. Nipah has been identified in a number of indigenous flying fox populations in Thailand. While no evidence of infection in domestic pigs or people has been found to date, pig farming is an active agricultural sector in Thailand and therefore could be a potential pathway for zoonotic disease transmission from the bat reservoirs. The disease, then, represents a potential zoonotic risk. To characterize the spatial habitat of flying fox populations along Thailand’s Central Plain, and to map potential contact zones between flying fox habitats, pig farms and human settlements, we conducted field observation, remote sensing, and ecological niche modeling to characterize flying fox colonies and their ecological neighborhoods. A Potential Surface Analysis was applied to map contact zones among local epizootic actors.

**Results:**

Flying fox colonies are found mainly on Thailand’s Central Plain, particularly in locations surrounded by bodies of water, vegetation, and safe havens such as Buddhist temples. High-risk areas for Nipah zoonosis in pigs include the agricultural ring around the Bangkok metropolitan region where the density of pig farms is high.

**Conclusions:**

Passive and active surveillance programs should be prioritized around Bangkok, particularly on farms with low biosecurity, close to water, and/or on which orchards are concomitantly grown. Integration of human and animal health surveillance should be pursued in these same areas. Such proactive planning would help conserve flying fox colonies and should help prevent zoonotic transmission of Nipah and other pathogens.

## Background

Habitat loss is the greatest threat to wildlife and biodiversity. The loss and fragmentation of wildlife habitats can lead to increasing contact among wildlife, domestic animals, and people, potentially leading to the emergence and spread of zoonotic diseases [1]. The Nipah virus (NiV) is one such pathogen. The novel RNA paramyxovirus (genus *Henipavirus*), closely related to Hendra virus, is named after the village Sungai Nipah in the State of Negeri Sembilan, Malaysia from which the virus was first isolated from a human patient in 1998 [2]. In humans, NiV causes Nipah virus infection, presenting a range of clinical outcomes, from asymptomatic infection to acute respiratory syndrome and fatal encephalitis [3].

Investigations of the origins of NiV identified the flying fox genus *Pteropus* to be a major reservoir [4,5]. Subclinical infections have been found in flying fox populations in Malaysia, Cambodia, Thailand and Madagascar [4-8]. Flying foxes are mammals, members of the *Pteropididae* or fruit bat family, and are the largest of all bats [9]. They are found throughout tropical and sub-tropical Asia and Australia and on islands of the Indian Ocean and the western Pacific [9]. *Pteropididae* play a crucial role in rainforest ecosystems [10]. They pollinate flowers and disperse seeds as they forage on the nectar and pollen of plants and on the fruits of rainforest trees and vines [10]. In Thailand, flying foxes are protected by the Wildlife Preservation and Protection Act, B.E. 2535 (1992), which forbids hunting protected wild animals and protects wildlife sanctuaries. A better understanding of the flying fox and its habitat preferences and dispersal would be a useful contribution to its conservation in Thailand. In addition, such an investigation should help efforts in better preventing potential disease transmission.

Work outside Thailand shows that in response to losses in its natural foraging areas, the adaptive *Pteropus* have turned to foraging in orchards, including those grown on pig farms where the NiV it carries are intermittently passed to pigs via urine or the contamination of partially-eaten fruit [4,5,11]. Investigation showed the virus to subsequently spill over from pigs to other animals and humans via respiratory droplets or close contact [2,12]. Pig farmers and workers exposed to respiratory illness and encephalitis in pigs were the first group of humans infected with the virus [13]. In 1999, abattoir workers in Singapore developed Nipah virus encephalitis [14]. Investigation showed direct contact with live pigs imported from Malaysia appeared to be the most important risk factor for those infections [15]. In contrast, a retrospective study of human cases in Bangladesh in 1999, the consumption of raw date palm sap proved one of the main risk factors of infection [16-18]. The result suggests NiV may have passed directly from bats to humans without an amplification host, as was apparently the case in Malaysia [11,12]. Human-to-human transmission was observed in several outbreaks in Bangladesh and India [18-20].

The situation of Nipah virus infection in Thailand showed that there has been no evidence of the viruses in domestic animals but they have been found in wildlife. Thailand’s National Institute of Animal Health (NIAH), the Department of Livestock Development (DLD)’s central laboratory, conducted a retrospective study of all specimens of swine interstitial pneumonia submitted during 1998 to 2001 using immunohisto-chemistry (IHC) technique [21]. All samples reported negative for NiV. Since 2002, The DLD has conducted a sero-surveillance of 4,000 – 5,000 samples of pig per year by using Modified ELISA technique. The pig blood samples have been collected in high pig density areas and bordering area of Thailand and Malaysia (south). Simultaneously, the veterinarians of the DLD have conducted clinical surveillance by investigating any suspected cases of NiV, they can consider to collect samples submitting for laboratory confirmation [22] but NiV has never been found so far [23]. On the other hand, the Molecular Biology Laboratory for Neurological Diseases, Chulalongkorn University Hospital conducted surveillance for NiV antibody by using enzyme immunoassay and for NiV by using the duplex reverse transcription–polymerase chain reaction (RT-PCR) in Thailand’s bat population during 2002–2004. The results showed 82 of 1,304 positives to NiV antibody and the tests for NiV presence in the urine and saliva of 12 bat species produced positives for 3 species of fruit bats (*P. hypomelanus, P. vampyrus*, and *P. lylei*) and 1 species of insect-eating bat (*Hipposideros larvatus*) with being a probable accidental case [7]. In only one species of flying fox (*P. lylei*) was NiV found in both saliva and urine. A longitudinal study subsequently conducted on *P. lylei* populations between 2005 to 2007 in Thailand showed that 2 NiV strains previously identified circulating in Malaysia and Bangladesh were found in the bat’s urine [24]. The study also highlighted a seasonal pattern with peaks between April and June, when viral RNA could be detected in urine. This seasonal pattern was associated with the observed fluctuation of population numbers, as May corresponds to the time of the year when young bats fledge [24].

The objectives of the present study were threefold. First, we aimed to describe the characteristics of the flying fox colonies and their neighborhoods in the central plain of Thailand (including central and eastern Thailand) from field observations, remote sensing (RS), and geographic information systems (GIS) data. Second, we aimed to predict the potential distribution of flying foxes in the study area using species distribution models (SDM). Finally, we aimed to map the areas where the three key elements of NiV ecology coincide, specifically flying fox habitat, human population, and pig farms, with the aim of informing NiV surveillance on the central plain of Thailand.

## Methods

### Characteristics of bat colonies and their vicinities

#### Field observations

**Figure 1:**
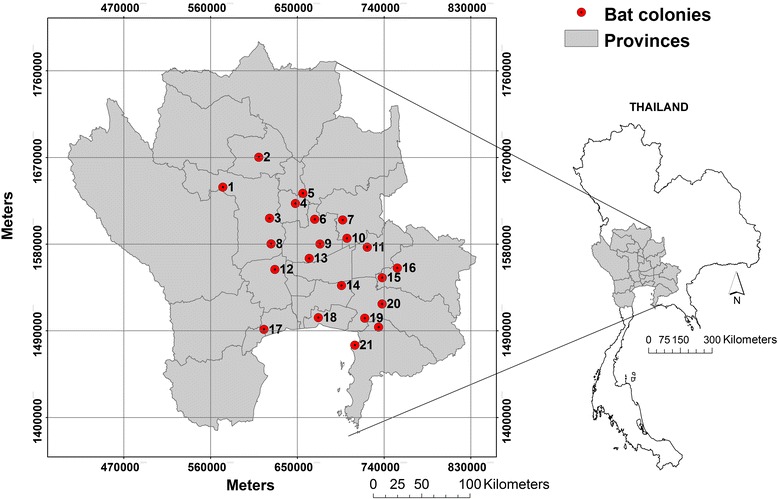
**Study area of flying fox colonies.** Study area covering 93,826.2 km^2^ of 23 provinces across western, central, and eastern Thailand (grey); 22 flying foxes’ colonies (red circles); comparing the size and locations of the study area and Thailand map (right).

The study area covered 23 provinces of western, central and eastern Thailand of a total area of 93,826 km^2^ (Figure 1). The distribution of flying foxes in central and eastern Thailand was studied in 2004 and 2011. Boonkird and Wanghongsa [25] surveyed the colony of flying foxes in central and eastern Thailand 2001–2004 and reported 16 sites in 10 provinces with 2 species of flying foxes: the Lyle’s flying fox (*P. lylei)* living in central Thailand and the Large flying fox or Greater flying fox (*P. vampyrus)* living along the coast of eastern Thailand. Sedsawai *et al*. [26] conducted a study of the distribution of flying foxes in central Thailand 2010–2011 and found 14 roosting sites within 10 provinces, including 10 previously reported and 4 newly discovered sites. Locations of bat colonies located in this area were obtained from these previous studies complemented by locations from field surveys by the Department of National Parks, Wildlife and Plant Conservation (DNP) conducted from March to August 2013. We surveyed each of those 22 bat colonies from June 2013 to January 2014 to verify the presence of flying foxes and to collect information on site characteristics for the roosting trees and their vicinities. We also estimated the margins of each colony with a hand-held GPS in order to delineate their spatial extent polygons.

#### Descriptive analyses

The GIS layer of the colonies was overlaid on other layers, including of bodies of water, human population density, elevation, and land cover. The vector map of permanent bodies of water was provided by the Ministry of Transportation. A human population density raster map at 100 m resolution was obtained from the Worldpop project [27]. We used the SRTM elevation database with 90 m spatial resolution produced by NASA [28].

A land cover map was developed using LANDSAT images with the Exelis VIS ENVI image processing software. Eleven scenes of the LANDSAT 7 Enhanced Thematic Mapper Plus (LANDSAT 7-ETM+) were used to cover the 23 provinces of the study area (path/row = 131/49-50, 130/49-51,129/49-51,128/49-51). The LANDSAT 7 ETM+ sensor has six optical spectral bands at 30 m spatial resolution and one panchromatic band at 15 m spatial resolution and a 16 day revisit cycle. We searched the LANDSAT image archive at the United States Geological Survey EROS Data Center (http://glovis.usgs.gov) and downloaded images with low cloud cover acquired in January 2014. All images were mosaicked and the minimum distance technique supervised classification method [29] was used to classify images into 4 land cover types most-related to flying fox habitat, including forest, irrigated vegetation, settlement/rainfed vegetation, and bodies of water. The regions of interest (ROIs) were built as classification training sets using ground truth data, 2D scatter plots, visible composition images, and spectral profiles. We evaluated the accuracy of the classes with 100 points per class of additional ground truth data and high-resolution data from Google Earth images for accuracy. Overall accuracy was 93%, with 98% accuracy for forest, 95% for irrigated vegetation, 91% for settlement/rainfed vegetation, and 86% for bodies of water, results considered acceptable and sufficient for the analysis.

For each bat colony polygon, we estimated summary descriptive statistics on the environment, geography, and anthropogenic variables. Specifically, we estimated the area of each colony, the distance from each colony to its nearest neighbor (another colony), the distance from each colony to the nearest body of water, the distance from each colony to the nearest temple, the average elevation within the colony, the average human population density, the proportion of irrigated vegetation land cover in a 10 km buffer around the colony, and the mean normalized difference vegetation index (NDVI) within the colony acquired from LANDSAT images. The vector map of Buddhist temples was provided by the Ministry of Transportation.

### Species distribution models

In this study, we used ensemble modeling (EM) (or consensus methods or ensemble forecasting) with the ‘dismo’ and ‘raster’ packages in R, which combines the predictions from several different statistical modeling techniques into a single prediction. Species distribution models (SDM) were initially used to map the ecological suitability for flying fox colonies across the study area. SDM can be used to predict the geographical distribution of species as a function of a series of spatial variables, as they relate species distribution data (occurrence or abundance in known locations) to information on the environmental and/or spatial characteristics of those locations [30]. They have been widely used both for describing patterns and making predictions across terrestrial, freshwater and marine ecosystems [30,31]. Flying fox colonies can occasionally move, and such modeling should allow inferring other areas to which colonies might move, even those from which they are presently absent. The variables used to build the models were selected according to field observations and the results of the descriptive analysis. This would allow, for example, to map areas where colonies are not present at the time of the study, but where the colonies may move in the future if they are too disturbed, or if their current habitat became degraded. The seven different SDM methods used in analyses include: Bioclim, Domain, generalized linear model (GLM), generalized additive model (GAM), maximum entropy model (Maxent), boosted regression tree (BRT), and random forests (RF). The Bioclim and Domain are presence-only modeling methods. Bioclim characterizes the occurrences that are located within the environmental hyper-space occupied by a species, whereas Domain is a distance-based method that assesses new locations in terms of their environmental similarity to locations of presence [32]. The GLM and GAM are presence-absence models based on the regression framework. The GLM is a generalization of ordinary least squares regression using maximum likelihood allowing the linear model to be related to the response variable via a logit link function. The GAM is an extension of the GLM, where the linear predictor is the sum of smoothing functions. It is more flexible and much as machine learning methods can fit very complex functions [33].

Maxent, BRT, and RF are machine learning methods using presence-absence data. Maxent, sometimes misleadingly referred to as presence-only methods, actually does require the use of background data [33]. It estimates species’ distributions by finding the distribution of maximum entropy (i.e. closest to uniform) subject to the constraint that the expected value of each environmental variable (or its transform and/or interactions) under the distribution matches its empirical average [34]. BRT combines the strengths of two algorithms, regression trees and boosting, creating a single best model from a large numbers of relatively simple models, each formed by a regression tree [35]. RF combines tree predictors such that each tree depends on the values of a random vector sampled independently and with the same distribution for all trees in the forest [36]. When compared with other methods, RF shows a very high accuracy, an ability to model complex interactions among predictors, and the flexibility to perform several types of statistical analysis [37]. The predictions of the seven SDM were then combined into a single ensemble model prediction by weighting each prediction by the performance of its source model, a procedure called ensemble modeling (EM) recognized as producing significantly more robust predictions than all the single models alone [38-42].

For three reasons, all our models were subject to 10 bootstraps. First, there was a very low proportion of positive samples in our data set, which can introduce bias into the logistic regression analysis framework. So for each trial we bootstrapped a different set of pseudo-absences [33,43]. Second, the bootstrapping also aims at preventing over-fitting. That is, we aim at avoiding modeling the noise rather than the main pattern in the data by assembling across a population of models trained with different subsets of data. Third, the pseudo-absences were distributed within a given distance of the presence sites. We wanted to bootstrap through different distance values. The 10 sets of absence data were randomly selected from the background and from 6–15 kilometers beyond the presence sites. Then each set was randomly selected again and divided into two parts equally: a model set used to train the model and a test set used to evaluate the models. These were then used as weights in combining the methods. Nine times the number of positives was randomly selected at each bootstrap to maintain 10% of the positive values of the outcome variable. This 10% ratio was chosen because previous studies compared the various prevalences across models and reported that GAM was not influenced by prevalence, whereas the accuracy increased up to an asymptote when the number of presences reached one tenth of the number of absences for GLM, BRT, and RF [33]. All predictor variables were simultaneously tested in the models.

The performance of the models was evaluated using the area under the curve (AUC) of the receiver operating characteristics (ROC) plots. AUC is a quantitative measure of the overall fit of models that varies from 0.5 (chance event) to 1.0 (perfect fit) [44]. Although AUC was recently criticized as an absolute measure of goodness of fit by many authors, it remains valuable in comparing the performances of several models tested on the same data set [32].

### Mapping the risk area of NiV

A Potential Surface Analysis (PSA) was applied to map the risk area of NiV by measuring the extent of the overlay between the factors that influence the risk of NiV infection, including potential flying fox habitats, and pig farm and human population densities. The PSA approach is somewhat simpler than other more complex knowledge-based approaches such as a Multi-Criteria Decision Analysis (MCDA) that can be employed to spatialize areas at risk [45]. However, MCDA methods require an extensive collection of knowledge on the important risk factors by experts, and at the present time, the knowledge of important cofactors that may be applicable to Thailand remains limited. In previous studies, the PSA method was used in similar conditions, where the knowledge of a particular outcome was limited. For instances; Ano *et al.* [46] estimated the drought risk area in the northeastern Thailand and used the result for managing water supply and Udomsap and Iamtrakul [47] studied the factors influencing the diversity of activities on Rachadamnoen Klang Avenue, Bangkok, which aimed to use the result in a planning process for maximizing efficiency of space usage and bringing economic enhancement to the local people and tourism. The PSA method ranks the spatial factors according to their importance using different weightings [48],

$$ S={W}_1{R}_1+{W}_2{R}_2+\left({W}_{\boldsymbol{n}}{R}_{\boldsymbol{n}}\right) $$

Where *S* represents a summation of scores, *W*_1_ − *W*_***n***_ , the weight of each factor according to its importance, and *R*_1_ − *R****n*** the rating score of each variable, which corresponds to its scaling into bins. For these maps we assumed two potential scenarios for *human infection:* 1) humans are directly infected by the virus from the bats, and 2) humans are infected through a pig intermediate host. The overlay corresponding to the first scenario was hence based on three factors: the flying fox distribution map, the distance to the flying fox colonies, and the house density in the sub-district. For the second scenario, the pig farm density map was added to the first three factors. For mapping the overlay of factors important to *pig infection,* we used three factors: the flying fox distribution map, distance to the flying fox colonies, and the pig farm density at the sub-district level.

The flying fox distribution map was obtained from the ensemble model described above, whose predicted values were divided into four bins according to their standard deviation (<0.5, 0.5-1.5, 1.5-2.5, and >2.5 of *σ*). The distance to the flying fox colonies was divided into bins corresponding to 5, 10, 20, 30, 50, 100, and 200 km. The pig farm density in the sub-district level was obtained from the 2010 surveys of the Department of Livestock Development (DLD), with the density values divided into 6 bins according to *σ*. The house density in the sub-district level was obtained from the Department of Provincial Administration, and the density values were divided into four bins according to their *σ*. We assigned initial rating and weighting scores to factors with values ranging from 0 to 9 (no risk to highest risk) based on literature and expert opinions [46-48] (Table 1). The layers were overlaid and analyzed by using the intersect tool. In each unit (intersected polygon), the summation score for each layer was summed. The mean and standard deviation were calculated from the summation scores of all units. The risk level was interpreted based on the summation score and the difference of mean $\left(\overline{x}\right)$ and standard deviation (*σ*). Risk was low if the summation score was less than $\overline{x}-\sigma$, moderate if the summation score ranged between $\overline{x}-\sigma$ and $\overline{x}-\sigma$, and high if the summation score was more than $\overline{x}-\sigma$.

**Table 1:** **Scores given for a Potential Surface Analysis (PSA)**

**Flying fox distribution zones (probability during 0 to1)**				**Distance to the flying foxes colonies (km)**				**Pig farm density (farm per km** ^**2**^ **)**				**House density (house per km** ^**2**^ **)**			
Scale	R	W	R*W	Scale	R	W	R*W	Scale	R	W	R*W	Scale	R	W	R*W
0.83x10^−3^ - 0.83x10^−2^	1	2	2	<5	9	1	9	0	0	3	0	0.13 – 907	2	3	6
0.84x10^−2^ - 0.14	2	2	4	5-10	8	1	9	0.9x10^−3^ -0.035	1	3	3	908-1968	4	3	12
0.15 - 0.27	3	2	6	10-20	7	1	7	0.036-0.88	2	3	6	1969-3028	3	3	9
0.28 - 0.87	4	2	8	20-30	5	1	5	0.89-1.72	3	3	9	3029-12510	1	3	3
				30-50	3	1	3	1.73-2.56	4	3	12				
				>50	1	1	7	2.57-16.55	5	3	15				

R = rating score W = weighting score.

Weighting and rating scores of 4 factors used to map the overlay between NiV hosts on the central plain of Thailand.

### Ethical considerations

This study was approved by the Research Committee of the Bureau of Disease Control and Veterinary Services, Department of Livestock Development (Permit Number: 0601/1325).

## Results

**Figure 2:**
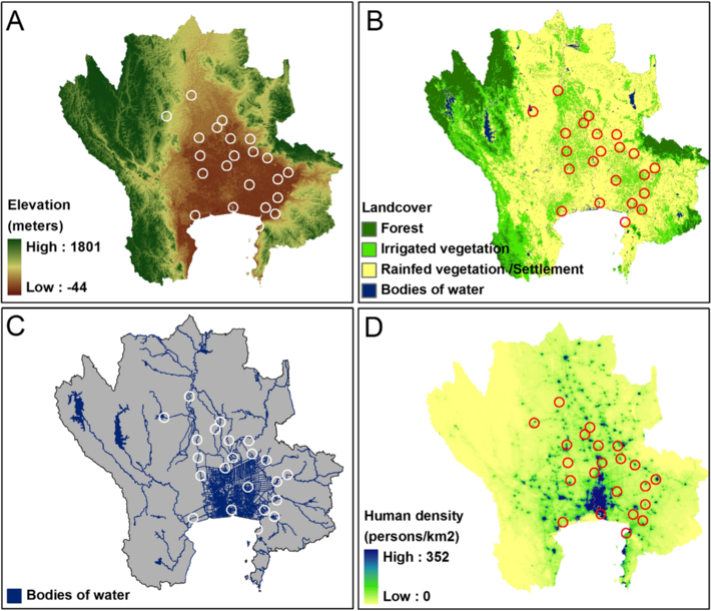
**Flying fox colonies compared to their environments.** Comparison among the locations of the flying foxes’ colonies (circle) and variables in the study area: elevation **(A)**; land cover **(B)**; bodies of water **(C)**; and human density **(D)**.

During field observation we observed flying foxes roosted on several types of trees: tamarind, coconut tree, bamboo (grass family), mangrove forests, and others (mostly members of evergreen forests) (Table 2). The colonies occupied a median area of 6,562 m^2^ (ranging 1,463-30,751 m^2^), and the median distance to the nearest neighbor colony was 23.2 km (ranging 12.5-57.7 km) (Table 3). Almost all colonies were located on the central plain (Figure 2A), with a median elevation of 9 m (ranging 5–65 m). The colonies clustered into 4 groups according to the type of roosting trees: 1) bamboo only; 2) mangrove forests only; 3) rubber trees only; and 4) various types of trees. We observed that while some trees failed to protect against sunlight, some colonies remained. Most colonies were located nearby Buddhist temples (median nearest distance 262 m, range 42–2704 m), with 13 of the 22 colonies roosting on trees located within the temple area (no. 3–7, 9–11, 13, 15, 16, 20 and 22). When overlaid over the land cover maps (Figure 2B), the majority of colonies were surrounded by irrigated vegetation covering 96% of the landscape within 10 km^2^, followed by settlement/rainfed vegetation (2.3%), bodies of water (1.5%), and forest (0.1%). Colonies were found on an island (no. 18) and riverside (no. 19), accessible to humans by boat alone. One colony was protected by the Wildlife Conservation Park (no. 1) and others located on private lands (no. 2, 8, 12, 14, 17, and 21). All colonies were located nearby bodies of water such as rivers, canals, ponds, and the sea (Figure 2C, median distance 120 m, range 30–4815 m). Some colonies were located in places with relatively high human population densities (Figure 2D), usually within Buddhist temples, where the number of tourists can be high (median population density of 232 people km^−2^, range 0–1307 people km^−2^), while one bat colony was located on an island uninhabited by humans. We observed that some colonies had moved away from their previously known sites. Colony no. 21 moved away from its old site to a new isolated site along the sea and colony no. 17 moved away from a site with numerous destroyed mangrove forest trees to an adjacent area.

**Table 2:** **Characteristics of the trees roosted by flying foxes**

**Group**	**Colony**	**Botanic description**
Group 1	8 and 12	Bamboo is generally found interspersed in many other types of forest and as a pioneer species. It is a fast growing species that easily colonizes disturbed forest sites, both natural and man-made. As such, and due to the logging excesses in Thailand in the past, many bamboo forests have become established in man-made disturbed sites [72].
Bamboo		
Group 2	17,18, 19, and 21	Trees in mangrove forest are evergreen species with a very dense forest floor. The roots of the trees are both for anchoring it in the soil and for breathing. This type of forest is found close to the seat of the mouth of major rivers where the sea washes ashore. The important tree species include the Kongklang (*Rhizophora* spp.), Prasak. The plants grow on the forest floor include the various types of sea grasses [73,74].
Mangrove forest		
Group 3	2,5, and 11	The rubber (*Dipterocarpus alatus*) is a tropical forest tree, of dense evergreen or mixed dense forests, common in Thailand, Cambodia, Laos and Vietnam. It is a medium-sized to fairly large tree of up to 40 m tall (sometimes more), bole tall, straight, cylindrical, branchless up to 20 m, up to 150 cm in diameter. Leaves narrowly ovate to ovate to elliptical-oblong, 9–25 cm x 3.5-15 cm, base cuneate to rounded, apex acute or shortly indistinctly acuminate [75].
Rubber		
Group 4	1,3,4,6,7,9,10,13,14, 15,16, 20 and 22	The various type of trees (mostly are in the Buddhist temple) composed of rubber (*Dipterocarpus spp*)*,* ficus tree *(Ficus spp),* bohhi tree (*Ficus religiosa* L.), bengal almond (*Terminalia catappa L.*), rain tree (*Samanea saman),* neem plant (*Azadirachta indica*), tamarind (Tamarindus indica Linn), sal tree (*Shorea robusta* Roxb.), bamboo (*Bambusa sp.*), coconut (*Cocos nucifera Linn*.), and others [75]. Most of trees are a medium-sized to fairly large tree of up to 40 m tall, which mostly found in the tropical evergreen forest which is distributed in all areas of Thailand. They are concentrated in pockets of high moisture such as valleys and close to water sources such as rivers, streams and mountains. A common characteristic of tropical evergreen forest is the appearance of a lush green vegetation all year round.
Various trees		

Characteristics of bat roosting trees of 22 flying fox colonies in the central plain of Thailand grouped by type of trees.

**Table 3:** **Descriptive statistics of the flying foxes’ colonies and vicinity**

**Statistics**	**Size (m** ^**2**^ **)**	**Distance from each colony**			**Elevation (m)**	**Human density (people/km** ^**2**^ **)**	**Land cover in 10 km. of radius (m** ^**2**^ **)**				**NDVI
		**To nearest neighbor (km)**	**To nearest water (m)**	**To nearest temple (m)**			**Forest**	**Irrigated vegetation**	**Settlement/rainfed vegetation**	**Bodies of water**	
Median	6562	23.2	120	262	9	232.07	259	246700	55840	3805	0.0119
*SD	8595	12.5	1442	683	13	339.39	4493	61194	47550	10017	0.0686
Minimum	1463	13.0	30	42	5	0	0	88680	14510	437	−0.1061
Maximum	30751	57.7	4815	2704	65	1307.15	16450	331800	158600	40470	0.1429

*Standard deviation **Normalized difference vegetation index.

Descriptive statistics of environmental, geographical, and anthropogenic factors of 22 colonies of flying fox on the central plain of Thailand.

**Figure 3:**
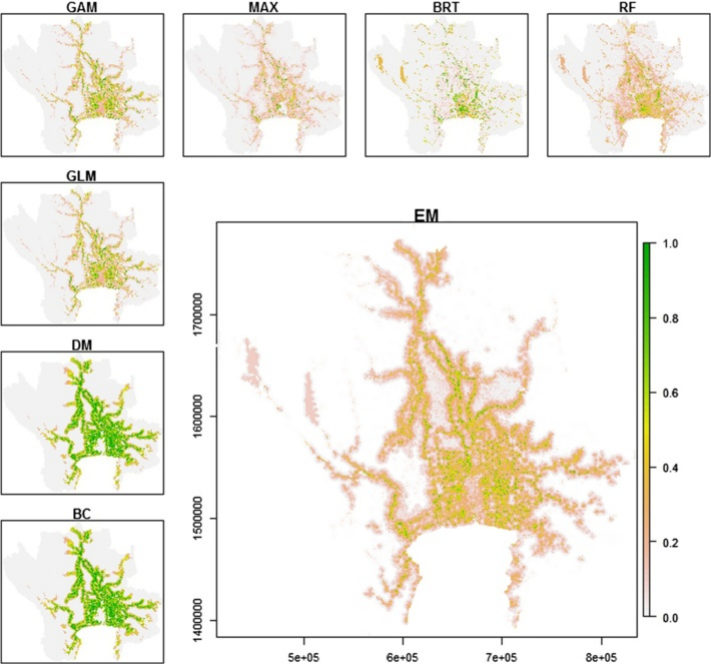
**Predicted suitability maps for flying fox colonies on the central plain of Thailand.** The maps explained by Bioclim (BC), Domain (DM), Generalized Linear Model (GLM), Generalized Additive Model (GAM), Maximum Entropy Model (MAX), Boosted Regression Tree (BRT), and Random Forest (RF). The large map shows the Ensemble model (EM) output obtained by combining the 7 SDMs weighted by their respective predictive performance.

**Figure 4:**
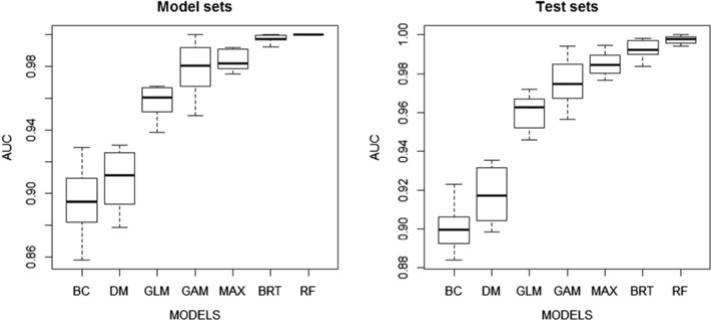
**The predictive performance of 7 species distribution models.** Box plots showing the predictive performance of 7 SDMs evaluated using the area under the curve (AUC) of ROC plots for the model sets (left) and test sets (right).

**Figure 5:**
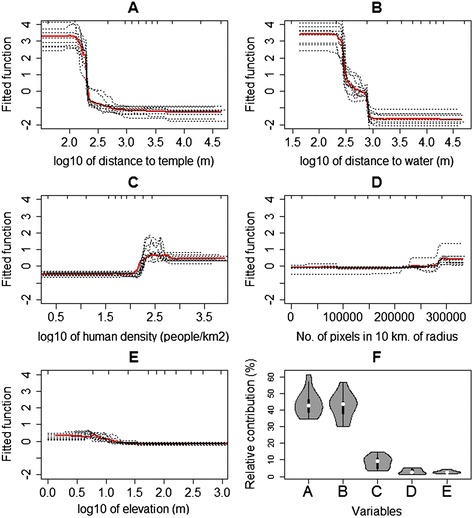
**Fitted functions and relative contributions of variables predicted by the BRT.** Partial dependence plots show the effect of a predictive variable on the response after accounting for the average effects of all other variables in the model: distance to water **(A)**; distance to temple **(B)**; human density **(C)**; amount of vegetation area within 10 km radius **(D)**; and elevation **(E)**. The relative contributions of each variable from the BRT is shown in **(F)**.

The predicted values obtained from the seven SDMs were combined as an ensemble model (EM) weighted by the predictive performance of each source model (Figure 3). All models captured the strong structuring effects of distance to rivers. The presence-only models (BC and DM) showed higher predicted values (more than 0.6) than that of the others in high-suitability areas. The model with the greatest AUC for evaluation was RF followed by BRT, Maxent, GAM, GLM, Domain, and Bioclim, respectively (Figure 4). The mean AUC of EM was 0.980 for model sets (ranged from 0.969-0.989) and 0.981 for test sets (ranged from 0.971-0.991). The effect of the predictive variable on predicted response (the fitted function) of the BRT model showed that the distance to temple, the distance to water, and elevation had a negative association and the area of vegetation within 10 km had a positive association with the presence of a colony (Figure 5). The human population density showed a positive association with the fitted function when human density was greater than 100 people per square kilometer and turned negative when the density was higher than 500 people per a square kilometer. The association remained steady when the density was higher than 800 people per square kilometer. The average relative contributions were 46% (35-62%) for the distance to a temple, 43% (31-55%) for the distance to the nearest body of water, 5.0% (2.6-8.7%) for the human density, 3.3% (0.6-6.5%) for vegetation area within 10 km of radius, and 3.2% (1.4-5.0%) for elevation.

**Figure 6:**
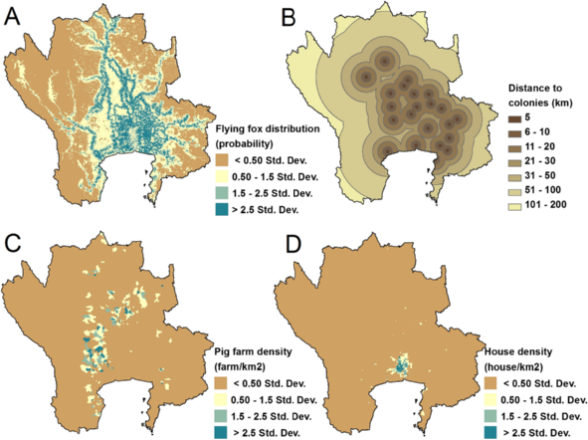
**Factors used in mapping NiV risk.** Maps of 4 factors used for analyzing the risk map of NiV in the central plain of Thailand: flying fox distribution map **(A)**; distance to the flying foxes colonies **(B)**; pig farm density at the sub-district level **(C)**; house density at the sub-district level **(D)**.

**Figure 7:**
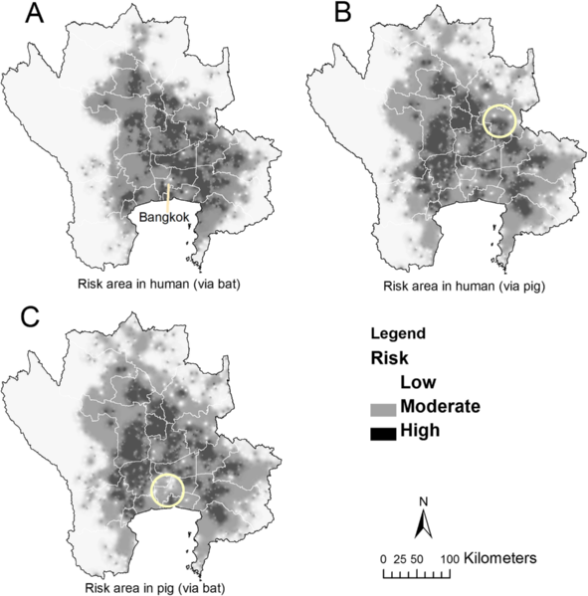
**Risk area of NiV in the central plain of Thailand.** Risk area of NiV produced by Potential Surface Analysis (PSA) based on i) flying fox distribution map, ii) distance to flying fox colonies, iii) house density and iv) pig farm density. The risk area of NiV for humans obtained from the first 3 factors **(A)**, from all 4 factors **(B)**, and the risk area of NiV for pigs produced by combining factors i, ii and iv **(C)**. The yellow circles show different risk areas between B and C. Risk was low if the summation score was less than $\overline{x}-\sigma$, moderate if the summation score was range between $\overline{x}-\sigma$ and $\overline{x}-\sigma$, and high if the summation score was more than $\overline{x}-\sigma$.

The overlay of potential surface maps corresponding to the first scenario under which *humans* are directly infected with NiV by bats show the higher-risk areas cover 6,199 km^2^ of 1,003 sub-districts, 159 districts, and 23 provinces and are mainly located to the north, northeast and east of Bangkok (Figures 6 and 7A). For the second scenario in which humans are infected via a pig reservoir, higher-risk areas cover 5,629 km^2^ of 653 sub-districts, 143 districts, and 23 provinces (Figure 7B). The higher-risk area of NiV in *pigs* cover 5,417 km^2^ of 607 sub-districts, 125 districts, and 23 provinces (Figure 7C). The two risk maps factoring in pig density looked very similar (Figure 7B & C). The higher-risk areas on both maps are located around the Bangkok metropolitan area, with environs to the west and north most affected. A slight difference in NiV risk levels between humans and pigs was observed in Bangkok, with greater risk for humans (Figure 7B) than for pigs (Figure 7C).

## Discussion

Our field observations indicated that flying foxes choose a variety of tree types, especially members of evergreen forests, for roosting, even if the trees no longer protect the bats from sunlight. The observations are supported by remote sensing, showing a normalized difference vegetation index acquired from January 2014 LANDSAT imagery with relatively low values at some of the roosting sites. This suggests flying foxes prefer evergreen forests to protect themselves from the sunlight while roosting, but also show a tolerance to trees damaged by bat urine and roosting [25]. We found roosting sites in relatively safe places, including Buddhist temples, islands, the Wildlife Conservation Park, and private lands, as marked by SDMs that included distance to a temple as an important predictor. Even as flying foxes are protected by the Wildlife Preservation and Protection Act, B.E. 2535 (1992), they are still threatened by human hunting, efforts to protect fruit orchards, and informal efforts at disease prevention. Most of the population is Buddhist (>90%) and would largely refrain from threatening animals in the vicinity of temples. Human density appears to correspond positively with roosting sites for temple communities but is negatively associated for the greatest densities in and around urban areas. Some bat colonies are located in private lands and study informants indicated landlords and/or the people in the local community around these sites had tried to protect the bats against hunters. Finally, the other colonies were located in isolated areas such as islands, riverside, and at seaside that are hard to reach by hunters. The Wildlife Conservation Park is closed off as a unit of wildlife conservation. Therefore, all bat colonies, across a variety of locales, were protected from hunters for an array of reasons, including cultural practices, ownership, local sentiment, and remoteness.

The distribution of flying fox colonies is dynamic and changes are observed over time. In 2004–2014, new colonies were observed and a few colonies moved away from their previously known sites [26]. Apart from disturbances caused by hunters, other factors may trigger colony migration. Disturbance by visitors or tourists is assumed to have caused colony no. 21 to move away from its old site to a new isolated site seaside. The roosting trees may have been damaged or killed by the flying foxes themselves by way of their urine and/or roosting [25]. Colony no. 17 moved away from a site with numerous destroyed mangrove forest trees to an adjacent area. Competition with other species using the same habitat could also play an important role as former bat colonies sites were observed colonized by large bird populations. Finally, colonies may move if their sizes increase beyond the capacity of a roosting site, if the foraging areas are reduced or too impacted by urban development, or in relation to the mating season [49]. Colony mobility supports the concept of mapping potentially suitable sites. Even should these sites be presently empty, they may be occupied in the near future.

The distance to bodies of water was found to be an important factor, both in the field and through statistical analysis. Rainho and Palmeirim [50] made similar observations in two cave-dwelling species (*Rhinolophus mehelyi* and *Miniopterus schreibersii*), for which proximity to a source of drinking water was an important factor. The Department of Environment of the Australian government also reported that flying foxes sites were usually found close to water [10]. Several studies indicated that bats lose a significant amount of water while they are roosting, especially under conditions of low relative humidity and high temperature [51-53]. Furthermore, lactating females need more frequent drinking than non-reproductive females [54]. Flying foxes may also need water for cooling down. Welbergen *et al*. [55] reported temperatures exceeding 42°C in January 2002 in New South Wales, Australia, causing the deaths of thousands of flying foxes from hyperthermia. The high temperature may lead flying foxes to dip their bellies into water to cool down [56]. The maximum temperatures in central Thailand in most months are above 30°C, with temperatures of 40°C commonly recorded in April [57]. As some roosting trees fail to protect bats from sunlight, the availability of nearby water may help those populations to resist the worst of the heat during the hottest months. Informants living nearby bat colonies suggested flying foxes may use bodies of water as landmarks for foraging. They reported flying foxes frequently flying along the river when they depart their roosting sites in the evening and when flying back along the river in the morning. Using water bodies as foraging landmarks was reported in insect-eating bats. For example, in the little brown bat, water bodies have been shown to be used as landmarks to help foraging on patches of insects found in abundance above rivers, streams, ponds, or lakes [58]. The association that we found between flying foxes and areas located in the lowland central plain, which is surrounded by vegetation, may also simply correspond to the extensive irrigation that allows greater vegetation than elsewhere, as observed in Australia [9].

Further studies, focusing on the distribution, ecology, behaviors, and disease status of flying foxes should be conducted in Thailand in the central region but also elsewhere. Although the foraging plants and some of the environmental factors associated with flying fox colonies have been reported in other countries, a follow-up should be pursued in Thailand and for its singular ecologies [9,59]. Such data would be useful for conserving flying fox populations and in disease prevention and surveillance. Flying fox movements, heat relief, water usage, and other behaviors should be more fully characterized as they are likely to have impacts upon transmission patterns. For instance, during the mating season, large aggregations of individuals migrating from different sites are observed, and, as documented in Arctic waterfowl, could potentially contribute to the spread of pathogens across bat and other populations [49,60].

The SDM maps converged with the observations discussed above, showing highly suitable areas for flying foxes mainly located along riversides, in river basins in the central plain, and in areas of moderate human population density. The number of known occurrences in our study was low (*n* = 22) and many studies note that small sample sizes can significantly reduce the predictive potential of models [31,61-63]. Several methods have been proposed to deal with the problem [33,64-66]. While some methods are more effective at predicting species’ distributions than others, no modeling method has proven to be the best in all situations. The ensemble modeling approach used in this study appears as a way to limit the potential influence of one particular modeling method, which was found to provide good results in previous studies [38,67,68]. However, we recognize that one of the limitations of our study may be the low sample size. One option for improvement might be to pool locations from wider areas and across countries, to have a larger sample size and sets of environmental conditions.

Even more challenging than mapping the suitability for a colony is to map the suitability for NiV infection. Generally, identifying risk factors associated with the spatial distribution of disease relies on disease distribution data that are used to quantify the effect of a set of explanatory variables on the spatial distribution of a particular disease outcome [69]. The outcome variable can be a count of disease events in a unit area or more simply a binary response indicating the presence or absence of disease at a given location. Each outcome can be used to map other areas sharing similar risk factors [69]. However, such an approach was not possible for NiV in Thailand since no case of NiV infection in human or pig has yet been found [23]. What we do have outside an etiological agent, in this case NiV, are susceptible hosts (bats, pigs and humans) and environments that connect hosts and the potential agent. By PSA we mapped areas where the virus’s documented reservoirs potentially coincide. The approach has not been used in epidemiological study but may be useful in the absence of disease data, as a way to spatialize disease surveillance and regionally plan livestock production. Even though it has not been used in epidemiological study and is not based on a formal statistical model, it remained useful in the present case of a disease that is absent (and hence provides no data to train a model) as a way to integrate different factors in a risk map that can inform further planning and disease surveillance in a context of very limited knowledge. A limitation of the approach is, however, the somewhat arbitrary choices on weights that are made along the process, that are defined in a more explicit and thorough way in using MCDA approaches. Ultimately, the spatial validity of both approaches could only be formally evaluated in retrospect, if NiV infection were eventually identified in the country.

## Conclusions

Broad-scale delineation of areas where three potential host types—bat, pig and human—are present could improve NiV surveillance strategy [70]. Indeed, in a context of limited financial support for animal disease surveillance systems, a more optimal use of resources could be implemented if active surveillance is targeted at higher-risk farms or areas [70]. One approach could circle around developing passive and active surveillance programs on pig farms of predicted risk, for example, with particular focus on farms of low biosecurity, nearby bodies of water, and/or hosting orchards as additional risk factors [11]. The surveillance program should be integrated with those for other diseases to reduce cost and manpower. Simultaneously, such surveillance efforts could be reinforced with enhanced communication on good farm management practices and public awareness campaigns.

In addition, preventing direct transmission of NiV from bats to humans could be adapted to the characteristic habitats identified in this study. For instance, it is apparent that flying foxes on the central plain of Thailand are found in particular conditions in spatial (e.g., distance to water, vegetation) and social terms (e.g., undisturbed environment and community). An active surveillance program could be conducted on the people who live closely to flying fox colonies. A new colony detected in 2011 (no. 17) is surrounded by commercial orchards, in particular coconut trees [26]. Testing NiV in a fresh coconut-palm sugar, usually produced by leaving a container on the trees overnight, may be useful for a focal study. In Bangladesh, sap harvesters were encouraged to use bamboo skirts on their trees to prevent contacts between fruit bats and raw date palm sap. Authorities educated locals to avoid drinking raw date palm sap or eat partially eaten fruit, and these efforts could be adapted for Thailand [71]. Finally, the central plain of Thailand is an area with intense farming activities, including pig husbandry, reflecting strongly the convergence across multiple risk models here. Surveillance programs in pigs and humans should be integrated to mutually increase their effectiveness.

## References

[1] Suzán G, Marcé E, Giermakowski JT, Armién B, Pascale J, Mills J (2008). The effect of habitat fragmentation and species diversity loss on hantavirus Prevalence in Panama. Ann N Y Acad Sci.

[2] Mohd Nor MN, Gan CH, Ong BL (2000). Nipah virus infection of pigs in peninsular Malaysia. Rev Sci Tech Int Off Epizoot.

[3] WHO | Nipah Virus (NiV) Infection [http://www.who.int/csr/disease/nipah/en/]

[4] Chua KB, Lek Koh C, Hooi PS, Wee KF, Khong JH, Chua BH (2002). Isolation of Nipah virus from Malaysian Island flying-foxes. Microbes Infect.

[5] Mohd Yob J, Hume F, Azmin Mohd R, Christopher M, Van Der Heide B, Paul R (2001). Nipah virus infection in bats (order Chiroptera) in peninsular Malaysia. Emergin Infect Dis.

[6] Reynes J-M, Counor D, Ong S, Faure C, Seng V, Molia S (2005). Nipah virus in Lyle’s flying foxes, Cambodia. Emerg Infect Dis.

[7] Wacharapluesadee S, Lumlertdacha B, Boongird K, Wanghongsa S, Chanhome L, Rollin P (2005). Bat Nipah Virus, Thailand. Emerg Infect Dis.

[8] Iehlé C, Razafitrimo G, Razainirina J, Andriaholinirina N, Goodman SM, Faure C (2007). Henipavirus and tioman virus antibodies in pteropodid bats, Madagascar. Emerg Infect Dis.

[9] DEPI - Flying-foxes [http://www.depi.vic.gov.au/environment-and-wildlife/wildlife/flying-foxes]

[10] Flying-foxes and national environmental law [http://www.environment.gov.au/node/16394]

[11] Chua K, Chua B, Wang C (2001). Anthropogenic deforestation, El Niño and the emergence of Nipah virus in Malaysia. Malays J Pathol.

[12] Tan K-S, Tan C-T, Goh K-J (1999). Epidemiological aspects of Nipah virus infection. Neurol J Southeast Asia.

[13] Chua KB, Goh KJ, Wong KT, Kamarulzaman A, Tan PSK, Ksiazek TG (1999). Fatal encephalitis due to Nipah virus among pig-farmers in Malaysia. Lancet.

[14] Paton NI, Leo YS, Zaki SR, Auchus AP, Lee KE, Ling AE (1999). Outbreak of Nipah-virus infection among abattoir workers in Singapore. Lancet.

[15] Chew MHL, Arguin PM, Shay DK, Goh K-T, Rollin PE, Shieh W-J (2000). Risk factors for Nipah virus infection among abattoir workers in Singapore. J Infect Dis.

[16] Hsu VP, Hossain MJ, Parashar UD, Ali MM, Ksiazek TG, Kuzmin I (2004). Nipah virus encephalitis reemergence, Bangladesh. Emerg Infect Dis.

[17] Luby S, Rahman M, Hossain M, Blum L, Husain M, Gurley E (2006). Foodborne transmission of Nipah virus, Bangladesh. Emerg Infect Dis.

[18] Luby SP, Gurley ES, Hossain MJ (2009). Transmission of human infection with Nipah virus. Clin Infect Dis.

[19] Luby SP, Hossain MJ, Gurley ES, Ahmed B-N, Banu S, Khan SU (2009). Recurrent zoonotic transmission of Nipah virus into humans, Bangladesh, 2001–2007. Emerg Infect Dis.

[20] Chadha MS, Comer JA, Lowe L, Rota PA, Rollin PE, Bellini WJ (2006). Nipah virus-associated encephalitis outbreak, Siliguri, India. Emerg Infect Dis.

[21] Pathchimasiri T, Kalpravidh W, Damrongwatanapokin S, Chantamaneechote T, Daniels P, Buranathai C: Immunohistochemistry Investigation of Nipah Virus : A Retrospective study in Thailand. In *The 11th International Symposium of the World Association of Veterinary Laboratory Diagnosticials and OIE Seminar on Biotechnology*. Thai Association of Veterinary Laboratory Diagnosticians; 2003:44–45.

[22] Department of Livestock Development (2012). Animal health in Thailand 2011.

[23] OIE World Animal Health Information System [http://www.oie.int/wahis_2/public/wahid.php/Countryinformation/Animalsituation]

[24] Wacharapluesadee S, Boongird K, Wanghongsa S, Ratanasetyuth N, Supavonwong P, Saengsen D (2010). A longitudinal study of the prevalence of Nipah virus in *Pteropus lylei* bats in Thailand: evidence for seasonal preference in disease transmission. Vector-Borne Zoonotic Dis.

[25] Boonkird K, Wanghongsa S (2004). On the population number and distribution fo flying foxes (Pteropus lylei) in central plain. Wildl Yearb.

[26] Sedwisai P, Changbunjong T, Chamsai T, Yongyuttawichai P, Sangkachai N, Weluwanarak T (2011). The distribution of flying fox (Pteropus spp.) in the central region of Thailand. J Appl Anim Sci.

[27] Gaughan AE, Stevens FR, Linard C, Jia P, Tatem AJ (2013). High resolution population distribution maps for Southeast Asia in 2010 and 2015. PLoS One.

[28] CGIAR-CSI SRTM 90 m DEM Digital Elevation Database [http://srtm.csi.cgiar.org/]

[29] Classification References (Using ENVI) | Exelis VIS Docs Center [http://www.exelisvis.com/docs/ClassificationReferences.html]

[30] Elith J, Leathwick JR (2009). Species distribution models: ecological explanation and prediction across space and time. Annu Rev Ecol Evol Syst.

[31] McPherson JM, Jetz W, Rogers DJ (2004). The effects of species’ range sizes on the accuracy of distribution models: ecological phenomenon or statistical artefact?: Species’ range and distribution model accuracy. J Appl Ecol.

[32] Elith J, Graham CH, Anderson RP, Dudík M, Ferrier S, Guisan A (2006). Novel methods improve prediction of species’ distributions from occurrence data. Ecography.

[33] Barbet-Massin M, Jiguet F, Albert CH, Thuiller W (2012). Selecting pseudo-absences for species distribution models: how, where and how many?. Methods Ecol Evol.

[34] Phillips SJ, Anderson RP, Schapire RE (2006). Maximum entropy modeling of species geographic distributions. Ecol Model.

[35] Elith J, Leathwick JR, Hastie T (2008). A working guide to boosted regression trees. J Anim Ecol.

[36] Breiman L (2001). Random forests. Machine learning, vol. 45.

[37] Cutler DR, Edwards TC, Beard KH, Cutler A, Hess KT, Gibson J (2007). Random forests for classificaiton in ecology. Ecology.

[38] Marmion M, Parviainen M, Luoto M, Heikkinen RK, Thuiller W (2009). Evaluation of consensus methods in predictive species distribution modelling. Divers Distrib.

[39] Araujo M, New M (2007). Ensemble forecasting of species distributions. Trends Ecol Evol.

[40] Robert J. Hijmans, Jane Elith: Species distribution modeling with R. 2013.

[41] Engler R, Waser LT, Zimmermann NE, Schaub M, Berdos S, Ginzler C (2013). Combining ensemble modeling and remote sensing for mapping individual tree species at high spatial resolution. For Ecol Manag.

[42] Thuiller W, Lafourcade B, Engler R, Araújo MB (2009). BIOMOD - a platform for ensemble forecasting of species distributions. Ecography.

[43] Augustin NH, Mugglestone MA, Buckland ST (1996). An autologistic model for the spatial distribution of wildlife. J Appl Ecol.

[44] Fielding AH, Bell JF (1997). A review of methods for the assessment of prediction errors in conservation presence/absence models. Environ Conserv.

[45] Stevens KB, Gilbert M, Pfeiffer DU (2013). Modeling habitat suitability for occurrence of highly pathogenic avian influenza virus H5N1 in domestic poultry in Asia: a spatial multicriteria decision analysis approach. Spat Spatio-Temporal Epidemiol.

[46] Ano T, Hormwichian R, Jitrapinate N, Compliew S, Kangrang A (2013). The estimation of drought risk area using potential surface analysis technique. UBU Eng J.

[47] Udomsap T, Iamtrakul P (2011). Accessibility improvement for district’s urban diversity: case study of Rachadamnoen klong avenue, Bangkok. J Soc Transp Traffic Stud JSTS.

[48] Nakya S, Leopairojna SK, Rangsiraksa L (2010). Use of satellite data and potential surface analysis for Urban expansion of Hua Hin Municipality, Prachuap Khiri Khan Province, Thailand. 31st Asian conference on remote sensing 2010. Volume 1.

[49] Eby P (1991). Seasonal movements of grey-headed flying-foxes, Pteropus poliocephalus (Chiroptera : Pteropodidae), from two maternity camps in northern New South Wales. Wildl Res.

[50] Rainho A, Palmeirim JM (2011). The importance of distance to resources in the spatial modelling of bat foraging habitat. PLoS One.

[51] Webb PI. The comparative ecophysiology of water balance in microchiropteran. 67;1995:203–218

[52] Webb PI, Speakman JR, Racey PA (2009). Evaporative water loss in two sympatric species of vespertilionid bat, Plecotus auritus and Myotis daubentoni: relation to foraging mode and implications for roost site selection. J Zool.

[53] Studier EH, O’Farrell MJ (1976). Biology of Myotis thysanodes and M. lucifugus (Chiroptera: Vespertilionidae)—III. Metabolism, heart rate, breathing rate, evaporative water loss and general energetics. Comp Biochem Physiol A Physiol.

[54] Adams RA, Hayes MA (2008). Water availability and successful lactation by bats as related to climate change in arid regions of western North America. J Anim Ecol.

[55] Welbergen JA, Klose SM, Markus N, Eby P (2008). Climate change and the effects of temperature extremes on Australian flying-foxes. Proc R Soc B Biol Sci.

[56] Killer climate: tens of thousands of flying foxes dead in a day [http://www.brisbanetimes.com.au/queensland/killer-climate-tens-of-thousands-of-flying-foxes-dead-in-a-day-20140225-33drr.html]

[57] Thai Meteorological Department [http://www.tmd.go.th/en/]

[58] Clare EL, Barber BR, Sweeney BW, Hebert PDN, Fenton MB (2011). Eating local: influences of habitat on the diet of little brown bats (Myotis lucifugus): molecular detection of variation in diet. Mol Ecol.

[59] Flying foxes | NSW Environment & Heritage [http://www.environment.nsw.gov.au/animals/flyingfoxes.htm]

[60] Olsen B (2006). Global patterns of influenza A virus in wild birds. Science.

[61] Stockwell DR, Peterson AT (2002). Effects of sample size on accuracy of species distribution models. Ecol Model.

[62] Chen H, Wood MD, Linstead C, Maltby E (2011). Uncertainty analysis in a GIS-based multi-criteria analysis tool for river catchment management. Environ Model Softw..

[63] Hirzel A, Guisan A (2002). Which is the optimal sampling strategy for habitat suitability modelling. Ecol Model.

[64] Segurado P, Araújo MB (2004). An evaluation of methods for modelling species distributions: methods for modelling species distributions. J Biogeogr.

[65] Engler R, Guisan A, Rechsteiner L (2004). An improved approach for predicting the distribution of rare and endangered species from occurrence and pseudo-absence data. J Appl Ecol.

[66] Hanberry BB, He HS, Palik BJ (2012). Pseudoabsence generation strategies for species distribution models. PLoS One.

[67] Lauzeral C, Grenouillet G, Brosse S (2012). Dealing with noisy absences to optimize species distribution models: an iterative ensemble modelling approach. PLoS One.

[68] Grenouillet G, Buisson L, Casajus N, Lek S (2011). Ensemble modelling of species distribution: the effects of geographical and environmental ranges. Ecography.

[69] Graham A, Atkinson P, Danson F (2004). Spatial analysis for epidemiology. Acta Trop.

[70] Katharina DS, Regula G, Hernandez J, Knopf L, Fuchs K, Morris RS, et al. Concepts for risk-based surveillance in the field of veterinary medicine and veterinary public health: Review of current approaches. BMC Health Serv Res 2006:6–20 10.1186/1472-6963-6-20 PMC1409776 16507106

[71] Khan SU, Gurley ES, Hossain MJ, Nahar N, Sharker MAY, Luby SP (2012). A randomized controlled trial of interventions to impede date palm sap contamination by bats to prevent Nipah virus transmission in Bangladesh. PLoS One.

[72] Forest Types in Thailand [http://wildlifethailand.com]

[73] Mangrove Forest Habitat in Tropical Thailand [http://www.kohphrathong.com/thailand_mangrove.html]

[74] Asia-Pacific Forestry Sector Outlook Study: Country Report - Thailand [http://www.fao.org/docrep/003/x2649e/X2649E03.htm]

[75] Biodiversity & Expert Database [http://www.bedo.or.th/lcdb/default.aspx]

